# Preretinal hemorrhage preventing subretinal involvement in multilayer macular hemorrhages: coincidence or plausible relationship?

**DOI:** 10.1186/s40942-021-00291-y

**Published:** 2021-03-16

**Authors:** Priscila A. Nascimento, Cristina N. Carbajo, Luisa S. M. Mendonça, Juliana T. Okimoto, Bogdan C. M. Barros, Julio Z. Abucham-Neto

**Affiliations:** 1 Department of Ophthalmology, Faculdade de Medicina Do ABC, Avenida Principe de Gales, 821, Santo André, SP 0906065 Brazil; 2 Instituto Suel Abujamra, São Paulo, SP Brazil

**Keywords:** Hemorrhages, Internal limiting membrane, Macular protection, Preretinal hemorrhage, Retinal hemorrhages, Subretinal hemorrhages, Premacular hemorrhage, Sub-ILM hemorrhage

## Abstract

**Background:**

To report four cases with interesting anatomical presentations of multilayer macular hemorrhages with preretinal hemorrhage possibly preventing subretinal involvement of the macular area.

**Cases presentation:**

Observational study of four patients presenting with macular hemorrhages.

**Results:**

Four patients with multilayer macular hemorrhage due to different causes, presented with a halo-shaped submacular hemorrhage coincident with the preretinal hemorrhage borders. After resolution, in all cases, the macular area underneath the preretinal hemorrhage was found to be spared.

**Conclusion:**

We hypothesized that an extensive preretinal hemorrhage can exert a mechanical force pushing the subretinal hemorrhage towards the periphery, consequently protecting the macular area.

## Background

Retinal hemorrhages are classified according to their topographic level in preretinal, intraretinal or subretinal. Certain diseases can manifest with multilayer hemorrhages, affecting all levels simultaneously, including macroaneurysm, Valsalva retinopathy, Terson syndrome, vascular tumors and hematological disorders [1–3]. It is known that submacular hemorrhage leads to retinal damage and permanent visual loss, which is thought to occur by three main mechanisms: barrier effect, tractional changes and toxic damage [4, 5]. The natural course of the disease, if left untreated, is severe, progressive, and often leads to irreversible vision loss [6].

Because visual prognosis depends on whether the macular area is affected by subretinal hemorrhage or not, the presence of massive preretinal hemorrhage should be considered an important factor in the assessment of retinal hemorrhages. In addition to its toxicity to the retina, it may conceal a subretinal hemorrhage, which would require early vitrectomy and tissue plasminogen activator (tPA) injection, or even a less invasive procedure, such as pneumatic displacement, to improve visual outcomes [3, 4, 6–8].

To illustrate the importance of adequate assessment of multilayered retinal hemorrhages and the challenging decision-making process surrounding early treatment of preretinal hemorrhage in order to achieve better macular visualization, we present four cases with interesting anatomical presentations of subretinal hemorrhage associated with preretinal hemorrhage, all of which suggest a future perspective regarding the treatment of this condition.

## Case reports

**Fig. 1:**
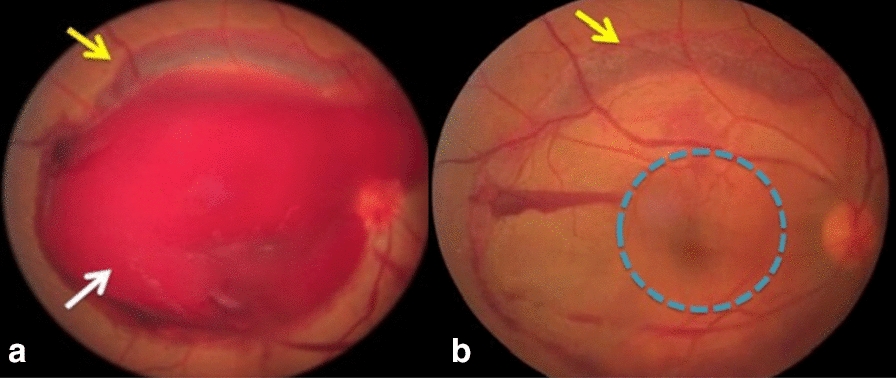
**a** Retinography on the left, revealing massive preretinal hemorrhage (white arrow) combined with surrounding subretinal hemorrhage (yellow arrow); **b** Retinography on the right, revealing the central macula free of subretinal hemorrhage (blue circle) after complete drainage of preretinal blood

*Case 1:* A 41-year-old man presented with low visual acuity (VA) of counting fingers (CF) in the right eye (OD) for two days due to Valsalva retinopathy. He presented with massive macular preretinal hemorrhage associated with scattered superior subretinal and intraretinal hemorrhages (Fig. 1a). Membranotomy was performed using an Nd: YAG laser. After the complete drainage of the preretinal blood, VA improved to 20/25, and it was possible to visualize the central macula, which was free of subretinal hemorrhage (Fig. 1b).

**Fig. 2:**
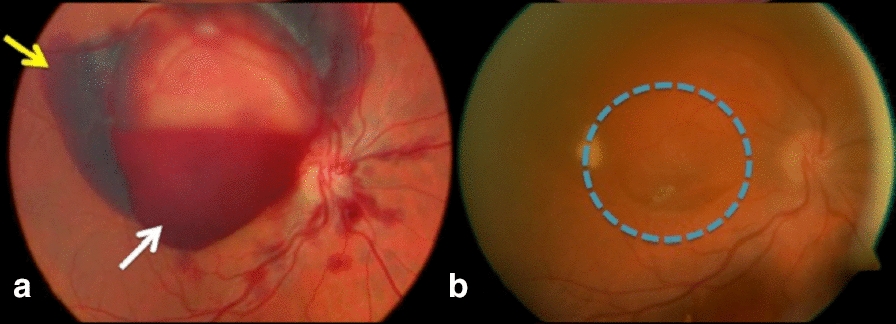
**a** Retinography on the left, showing a subretinal hemorrhage (yellow arrow) around the preretinal blood (white arrow); **b** Retinography on the right, acquired after treatment with membranotomy, demonstrating macula free of subretinal blood (blue circle)

*Case 2:* A 34-year-old woman presented with low VA of CF in the OD and 20/40 in the left eye (OS) shortly after a non-complicated abdominal liposuction surgery under peridural anesthesia. Extensive macular preretinal hemorrhage was observed in the OD, surrounded by subretinal hemorrhage, in addition to scattered peripapillary intraretinal hemorrhages (Fig. 2a). The OS presented only scattered intraretinal hemorrhages. Membranotomy treatment with Nd: YAG laser was performed in the OD, allowing the observation that the macula was free of subretinal hemorrhage, followed by improvement in VA to 20/60 in the OD and 20/20 in the OS (Fig. 2b).

**Fig. 3:**
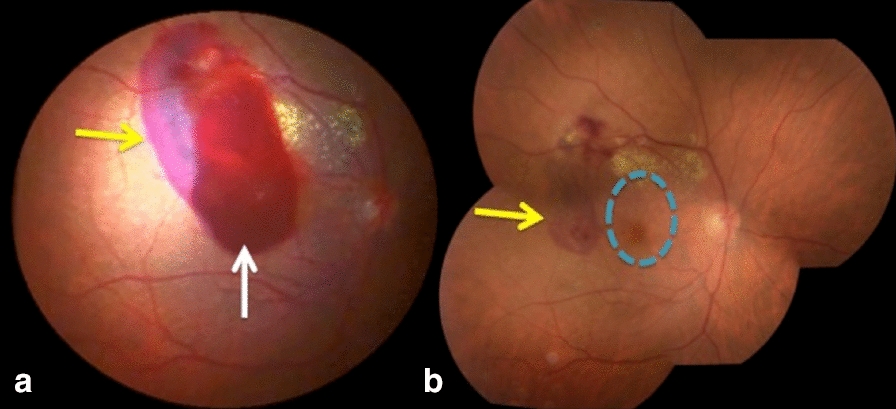
**a** Retinography on the left. Note the preretinal hemorrhage (white arrow) combined with surrounding subretinal hemorrhage (yellow arrow), and the presence of hard exudates; **b** Retinography on the right. The macula appears spared (blue circle) after drainage of the preretinal blood with pars plana vitrectomy, with surrounding subretinal hemorrhage (yellow arrow)

*Case 3:* A 64-year-old woman presented with abrupt-onset low VA in the OD of CF. She had past ophthalmological history of low VA (hand motion) in the OS for 5 years due to polypoidal choroidal vasculopathy. In the right eye, she presented with preretinal hemorrhage the superior temporal arcade topography, extending to the macular region with adjacent hard exudates and subretinal hemorrhage in its temporal margin (Fig. 3a). The left eye showed an extensive disciform scar at the posterior pole. Posterior pars plana vitrectomy and internal limiting membrane (ILM) peeling were performed in the right eye, confirming the intraoperative diagnosis of ruptured retinal arterial macroaneurysm. After treatment, final VA was 20/30 in the OD (Fig. 3b).

**Fig. 4:**
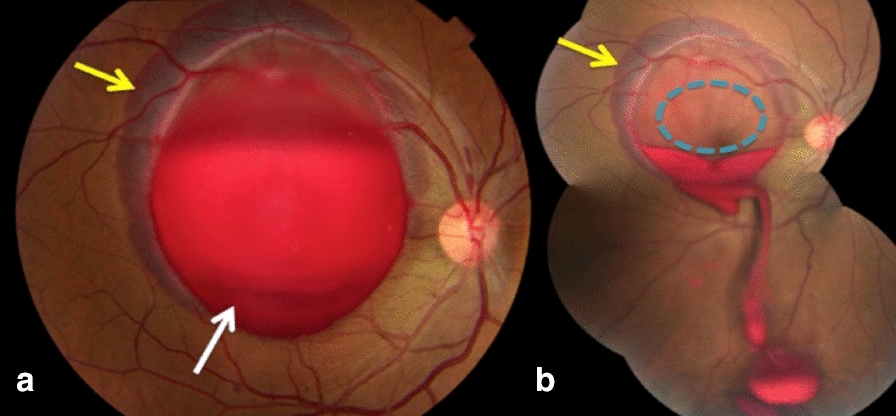
**a** Retinography on the right revealing preretinal hemorrhage (white arrow) combined with surrounding subretinal hemorrhage (yellow arrow); **b** Retinography on the right. Note the maintenance of the subretinal hemorrhage (yellow arrow), despite the clear macula after drainage of the preretinal blood (blue circle)

*Case 4:* A 39-year-old man presented with sudden low VA of CF in the OD after sexual intercourse, due to preretinal hemorrhage at the posterior pole, combined with subretinal hemorrhage in nearly 360° of its margins (Fig. 4a). Nd: YAG laser was used to treat the OD in the inferior portion of the hyaloid, achieving best-corrected VA of 20/20 after treatment, and the possibility of designating the macular status as free of subretinal involvement (Fig. 4b).

## Discussion

Subretinal hemorrhage leads to unclear visual prognosis, directly depending on the time to intervention, especially in cases with submacular involvement. Malik [3] suggested that the retinal damage and permanent visual loss secondary to hemorrhage is thought to occur by three main mechanisms: a barrier effect, tractional changes and toxic damage, all of which are time-dependent. Adding to this theory, Glatt and Machemer [5] demonstrated irreversible retinal injury with photoreceptor edema and pyknosis of the outer nuclear layer within 24 h after subretinal injections of autologous blood in rabbits. After 7 days, the authors found more severe degeneration with shearing of photoreceptor outer segments and retinal pigment epithelium (RPE) cells showing shortened apical microvilli and mitochondrial distortion. After 14 days, there was outer retina and RPE atrophy and disorganization with vacuolization, accompanied by an influx of phagocytic cells.

In many diseases, subretinal hemorrhages are associated with preretinal hemorrhages, in a multilayered involvement of the macula. This can lead to the occurrence of epiretinal membranes [2] and even more concerning, can impair the visualization and assessment of the subretinal hemorrhage. For this reason, prompt removal of the preretinal hemorrhage is important [6]. Preretinal hemorrhage can be treated with several strategies [7], including Nd: YAG laser hyaloidotomy [9], pneumatic displacement by intravitreal injection of gas and tissue plasminogen activator [8] and pars plana vitrectomy [10]. Waiting for the spontaneous reabsorption is also a possible approach; however, the time course is unclear, even more so if the status of the subretinal area is unknown.

**Fig. 5:**
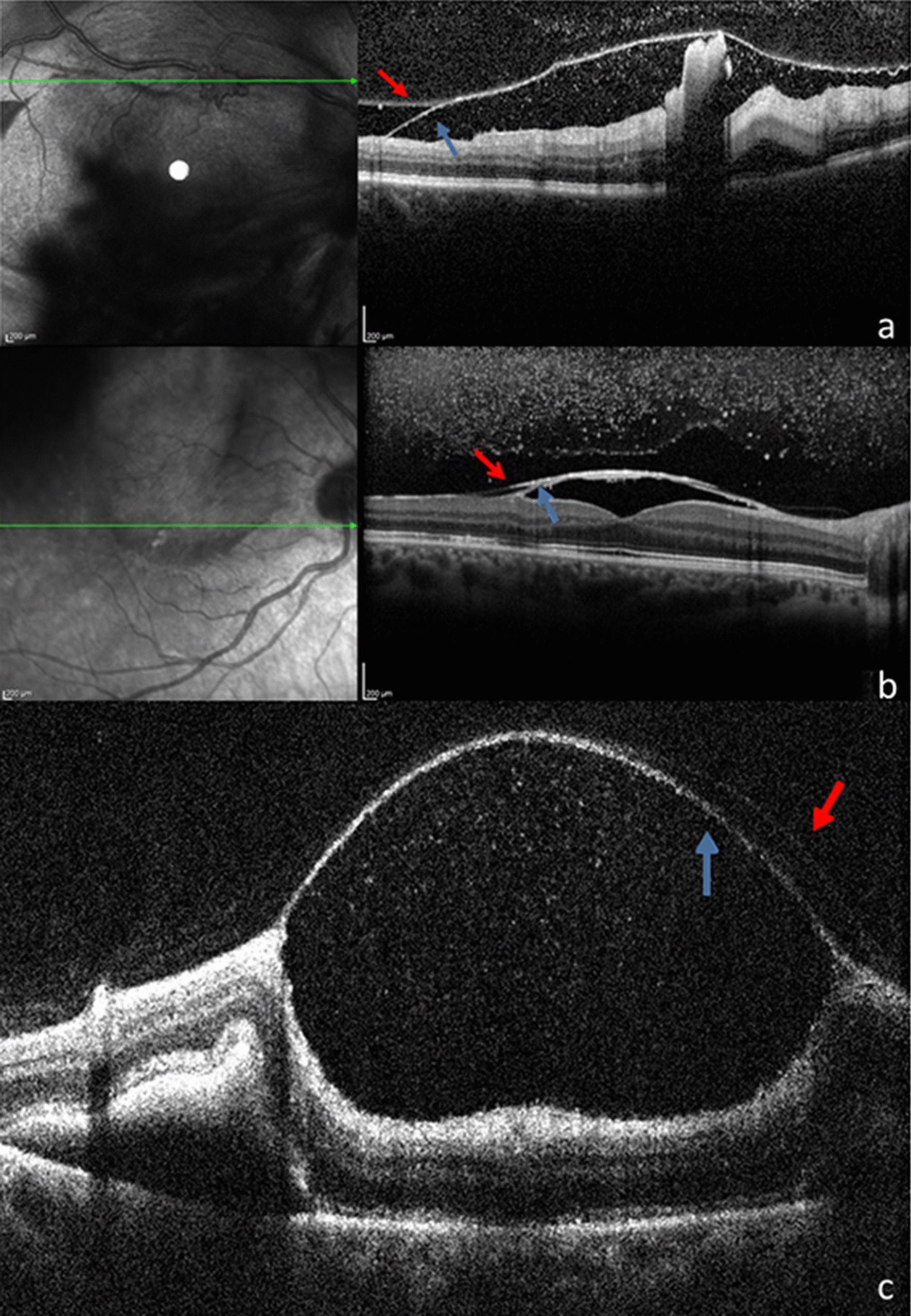
**a**, **b** and **c** SD-OCT acquired after treatment, correspondent to case 1, 3 and 4 respectively, demonstrating preretinal hemorrhage location under the internal limiting membrane (blue arrow) and its separation from the posterior hyaloid (red arrow)

For all these reasons, establishing an early diagnosis is important in order to institute prompt treatment of subretinal hemorrhage and improve visual prognosis. There remains a question as to whether any sign would help in predicting subretinal involvement in cases with extensive preretinal macular hemorrhage. The cases reported in this study revealed a peculiarity: a halo-shaped submacular hemorrhage, coinciding with the boundaries of the preretinal hemorrhage. Based on this finding, we hypothesized that if the preretinal hemorrhage [specifically under internal limiting membrane (Fig. 5a–c)] exerts a mechanical force, pushing the subretinal hemorrhage towards the peripheral region, it might protect macular function. The lack of foveal involvement underneath the pre-existing preretinal hemorrhage that was found after treatment, as well as the satisfactory final visual acuity in all cases, support our hypothesis.

Stopa et al. [7] studied the mechanism of action of intravitreal gas for displacement of subretinal hemorrhages. They found that submacular hemorrhage remains static because of the contraposition of thrust forces (generated by the vitreous action on the hemorrhage) and gravity. Intravitreal pneumatic injection reduces the thrust force, leading to the displacement of the hemorrhage by the action of gravity. The question is raised as to whether preretinal hemorrhage acts similarly to an intravitreal gas injection, only spontaneous and instantaneously. According to our theory, this would be the case. The blood drawn into the internal limiting membrane space creates a superficial tension that is greater than vitreous pressure, possibly pushing subretinal blood centrifugally towards the periphery, protecting the macular area.

Nakamura et al. [10] reported two similar cases that are particularly interesting, where, following removal of sub-ILM hemorrhage, there was mild subretinal hemorrhage coinciding with the site of the sub-ILM hemorrhage, and dense doughnut-shaped hemorrhage in the margins of the preretinal hemorrhage. It appeared as though the sub-ILM hemorrhage had pushed out the blood beneath the retina.

In addition to these cases, we found in the literature a report of four cases of preretinal hemorrhage treated with Nd: YAG Laser, one of which presented the same findings described in our series: preretinal hemorrhage surrounded by subretinal hemorrhage that, after treatment, showed a spared macula and subretinal hemorrhage coincident with the preretinal hemorrhage borders [2].

In conclusion, in cases of extensive preretinal hemorrhage where there is a halo-shaped subretinal hemorrhage circumjacent, it is probable that the macular area is spared of subretinal hemorrhage. Further studies are needed in order to prove this theory, and to test whether an expectant approach would be appropriate in such cases.

## Data Availability

All data generated or analysed during this study are included in this published article.

## Abbreviations

tPA: Tissue plasminogen activator
VA: Visual acuity
CF: Counting fingers
OD: Right eye
OS: Left eye
ILM: Internal limiting membrane
RPE: Retinal pigment epithelium
